# Division of labor in bacteria

**DOI:** 10.7554/eLife.38578

**Published:** 2018-06-29

**Authors:** Alma Dal Co, Charlotte Brannon, Martin Ackermann

**Affiliations:** 1 Department of Environmental Systems SciencesETH ZurichZurichSwitzerland; 2 Department of Environmental MicrobiologyEawagZurichSwitzerland; 3 Department of Molecular Cellular and Developmental BiologyYale UniversityNew Haven, CTUnited States

**Keywords:** division of labor, Bacillus subtilis, acetate, population, fermentation, stochastic gene expression, *B. subtilis*

## Abstract

The emergence of subpopulations that perform distinct metabolic roles has been observed in populations of genetically identical bacteria.

**Related research article** Rosenthal AZ, Qi Y, Hormoz S, Park J, Li SH, Elowitz MB. 2018. Metabolic interactions between dynamic bacterial subpopulations. *eLife* **7**:e33099. doi: 10.7554/eLife.33099

Why do we have so many different types of cells in our body? A plausible explanation is that a single cell can only perform a limited number of roles at the same time. As a consequence, our body consists of more than 200 clearly distinguishable types of cells (Heintzman et al., 2009).

But what about smaller life forms such as bacteria? These microbes often form communities of genetically identical, or clonal, cells that can collectively activate genes or regulate metabolic processes. It remains unclear, however, whether each single cell performs all the roles observed at the population level, or whether cells specialize on a subset of these functions. Although a division of labor has been suspected, evidence remains scant (Nikolic et al., 2013; de Lorenzo et al., 2015; Ackermann, 2015). Now, in eLife, Michael Elowitz of the California Institute of Technology and colleagues – including Adam Rosenthal as first author – report how a clonal population of *Bacillus subtilis* bacteria divides into two subpopulations with distinct roles (Rosenthal et al., 2018).

The two groups of bacteria likely arose from a phenomenon known as stochastic gene expression. This happens when the randomness associated with the transcription and translation of genes is amplified by the cell’s regulatory network, and results in cells with identical genomes having different traits or phenotypes (Elowitz et al., 2002). While many cases of stochastic gene expression have been described in the last decade, two aspects set this study apart from previous work (Kaern et al., 2005): first, the two subpopulations engaged in metabolic interactions; second, the composition of the population changed dynamically over time (Figure 1).

**Figure 1.**
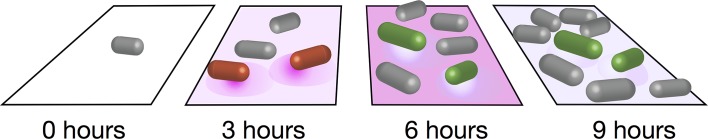
Distinct metabolic pathways in a population of bacteria. Schematic showing the two subpopulations of *B. subtilis* seen in the experiments of Rosenthal et al. At the start of the experiment (left) an individual bacterium (grey) grown on glucose and malate divides to give rise to a clonal population. After three hours, some of the cells (shown in red) start to secrete acetate (purple shadow). After six hours, acetate has accumulated to a toxic level, and a second phenotypic subpopulation emerges: the bacteria in this second subpopulation (green) take up the acetate and convert it to acetoin, which is nontoxic. After nine hours, acetate has dropped to a level that is non-toxic.

Rosenthal et al. – who are based at Caltech and Princeton University – grew several populations of *B. subtilis* on two sugars, glucose and malate, and observed that some bacteria did not metabolize these sugars completely, but instead began secreting acetate, a metabolic intermediate. Secreting acetate can help bacteria to grow more quickly, but it can also accumulate to toxic levels (Pfeiffer et al., 2001). The researchers found that this toxicity was remediated by a small subpopulation of bacteria that started to convert acetate to acetoin, which is harmless, thus enabling the population to grow in a detoxified environment. These acetate-consuming cells only emerged after the acetate-producing cells arose, as a direct response to the accumulating toxicity. This demonstrates that individual cells in a population of genetically identical bacteria can activate alternative metabolic pathways that affect the population as a whole.

The study of Rosenthal et al. raises an intriguing question: does a metabolic division of labor happen often in clonal populations of bacteria? There are reasons to assume that it would be beneficial and therefore potentially widespread. For example, recent studies suggest that bacteria can divide up to 20% faster if they trade certain cellular 'building blocks' with other bacterial species, instead of producing them on their own (Pande et al., 2014). Thus, one might expect the cells in clonal populations to differentiate and specialize in order to make the most of their labor. Moreover, bacterial communities often live on surfaces, where they can reach high densities (Hall-Stoodley et al., 2004). Such conditions could favor metabolic differentiation and the division of labor.

Microbes play crucial roles in our lives, from cycling elements on our planet to shaping health and disease. If metabolic division of labor is widespread in bacterial populations, this finding could have a profound impact on biology. In fact, many widely-studied metabolic pathways in bacteria could actually be the result of a group effort that no single cell could perform alone.

Although the degree of cell differentiation in the human body most likely exceeds that in bacterial populations, these two systems may nevertheless share an interesting feature. In both cases, the properties of the system emerge from interactions between cells that are genetically identical but phenotypically different. It is intriguing to think that these two very different life forms may have found a common solution to a general problem: if a single cell cannot perform more than a certain number of roles, a collection of cells can organize itself to ensure that all functions are still accomplished.
